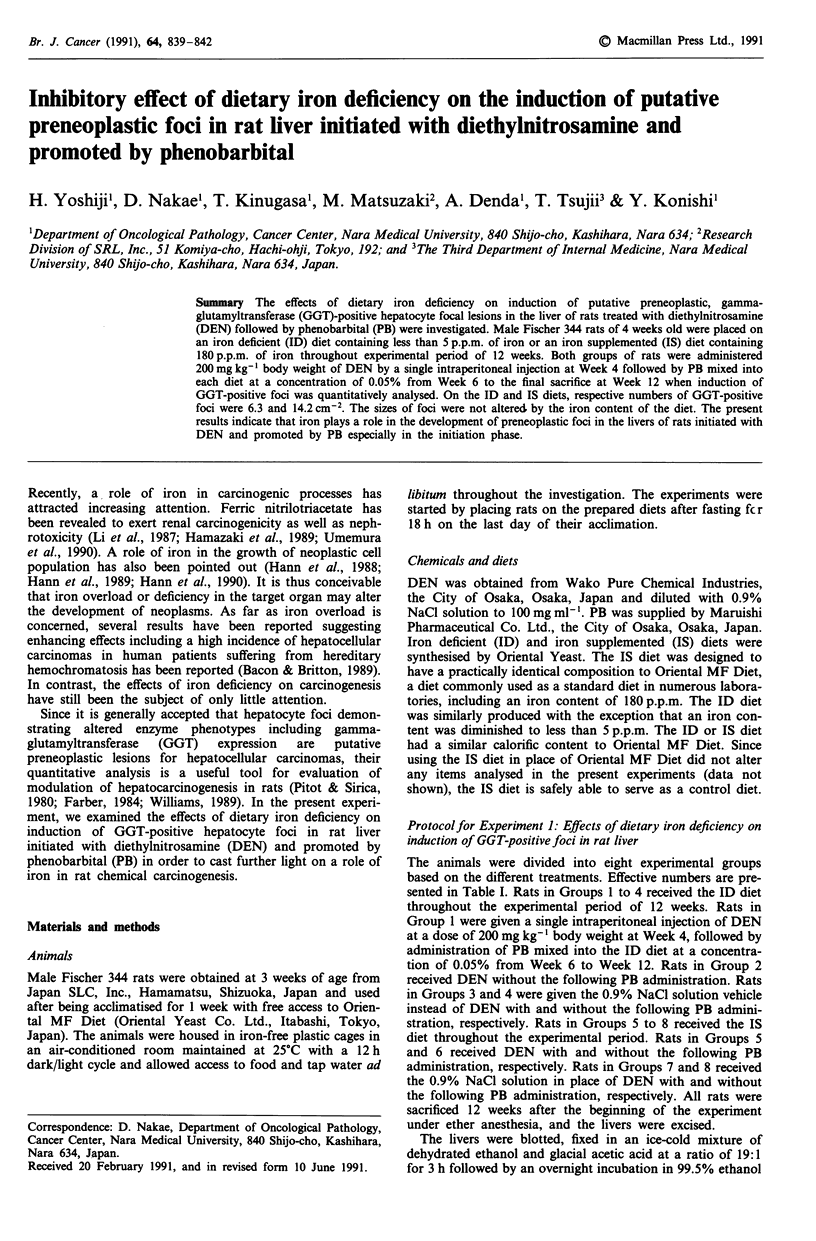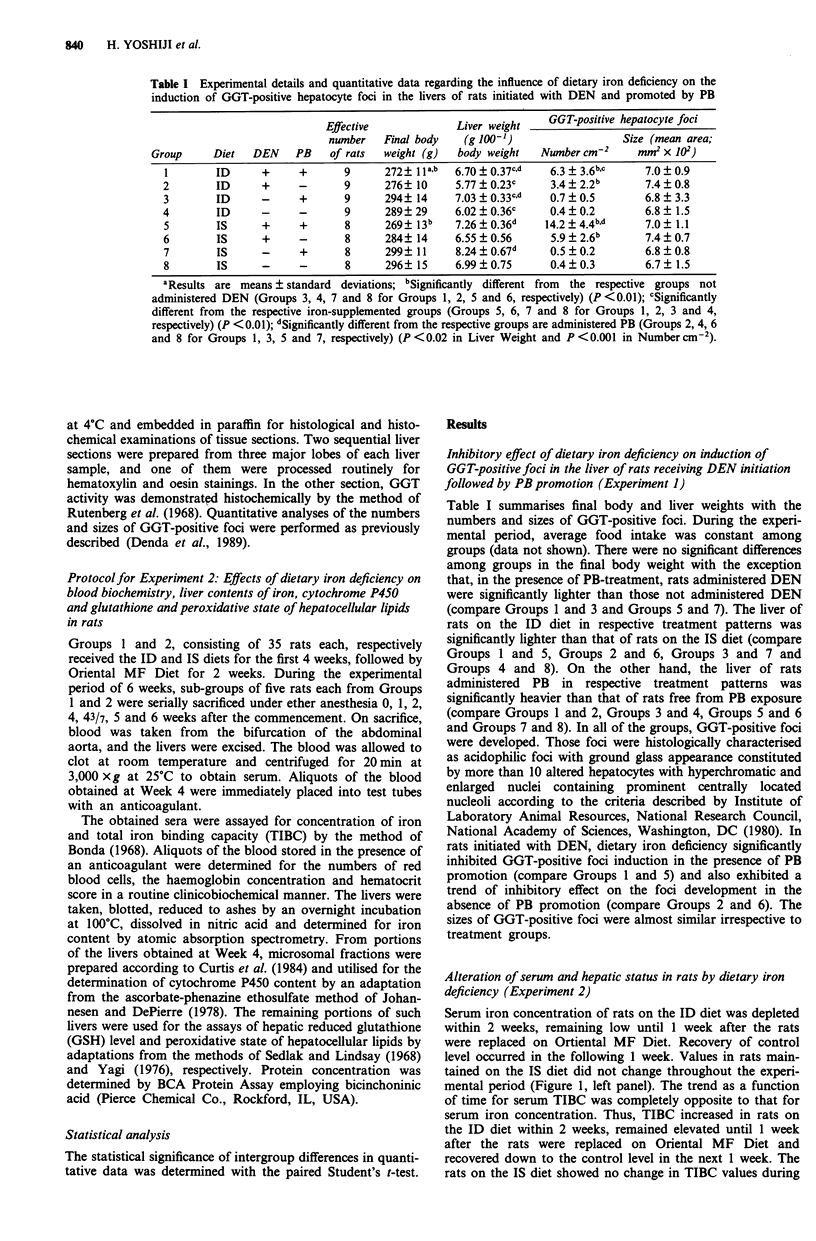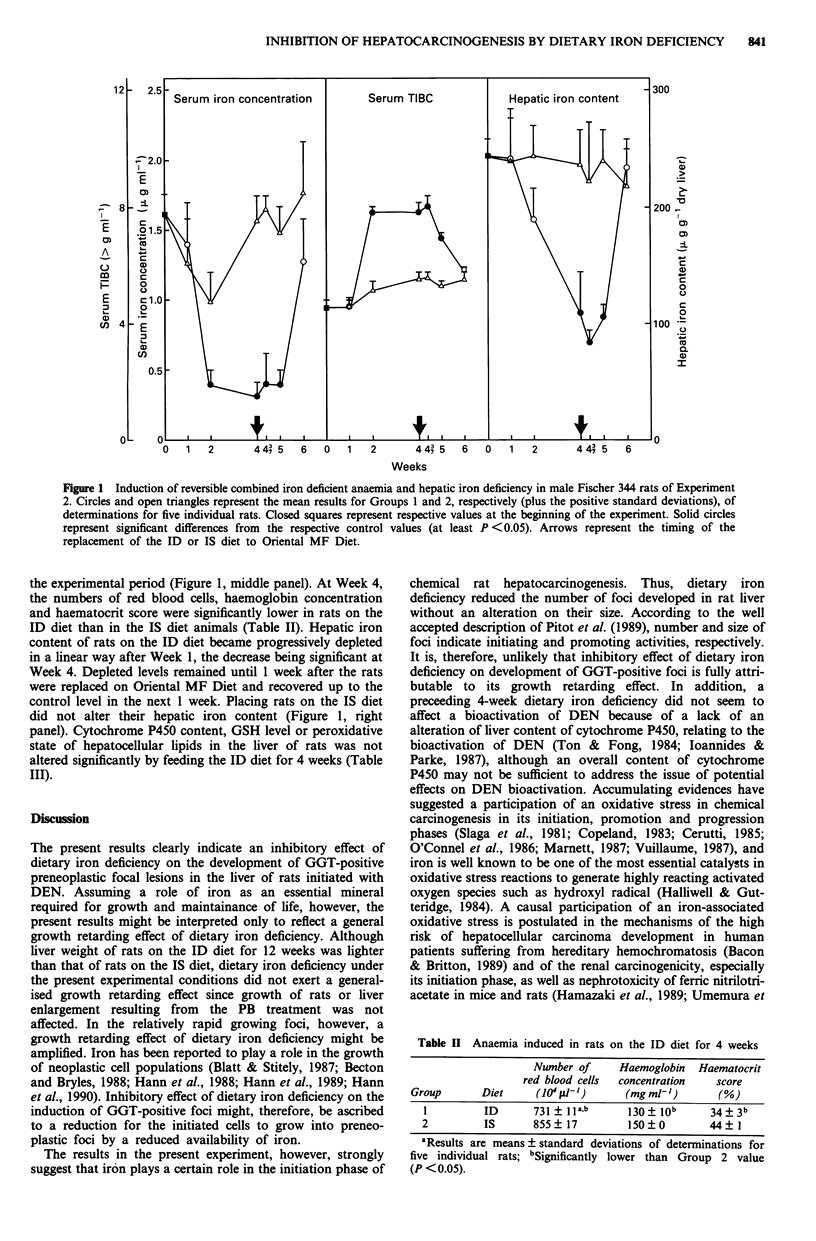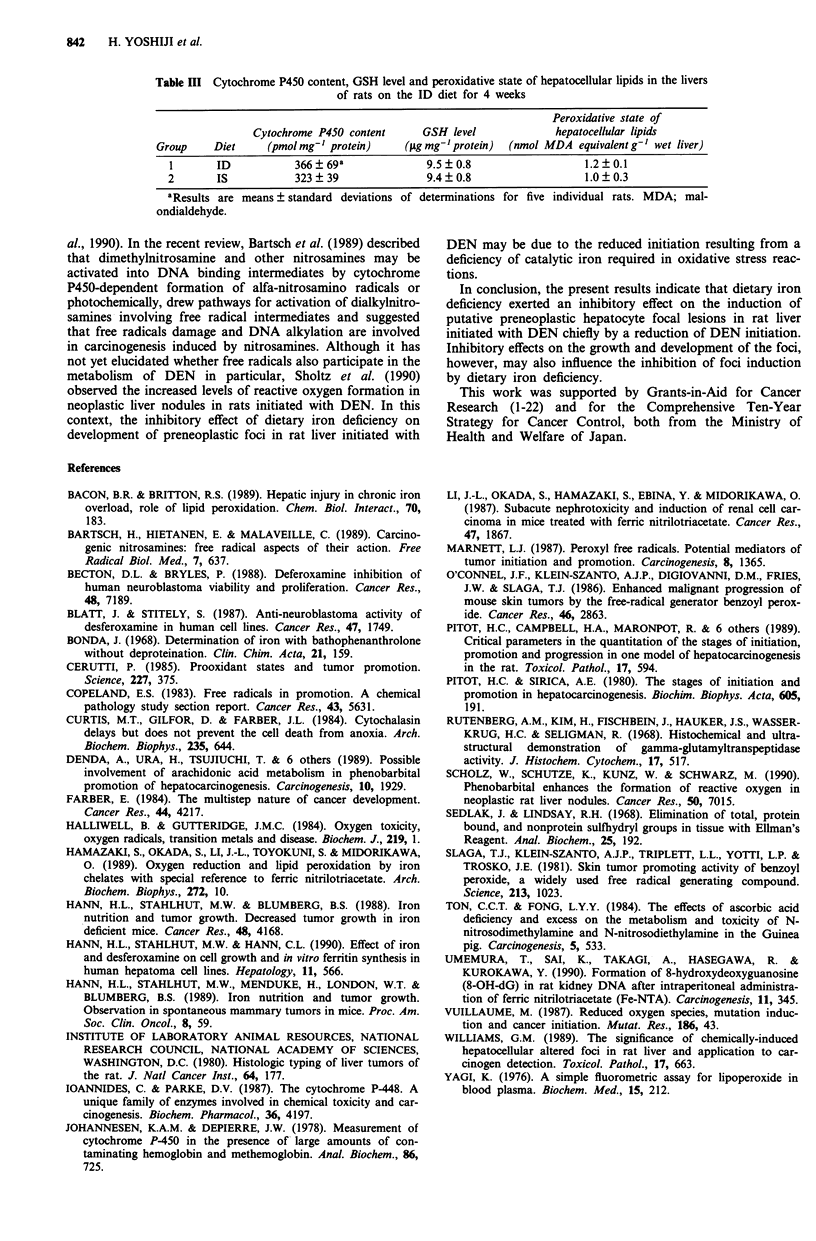# Inhibitory effect of dietary iron deficiency on the induction of putative preneoplastic foci in rat liver initiated with diethylnitrosamine and promoted by phenobarbital.

**DOI:** 10.1038/bjc.1991.410

**Published:** 1991-11

**Authors:** H. Yoshiji, D. Nakae, T. Kinugasa, M. Matsuzaki, A. Denda, T. Tsujii, Y. Konishi

**Affiliations:** Department of Oncological Pathology, Nara Medical University, Japan.

## Abstract

The effects of dietary iron deficiency on induction of putative preneoplastic, gamma-glutamyltransferase (GGT)-positive hepatocyte focal lesions in the liver of rats treated with diethylnitrosamine (DEN) followed by phenobarbital (PB) were investigated. Male Fischer 344 rats of 4 weeks old were placed on an iron deficient (ID) diet containing less than 5 p.p.m. of iron or an iron supplemented (IS) diet containing 180 p.p.m. of iron throughout experimental period of 12 weeks. Both groups of rats were administered 200 mg kg-1 body weight of DEN by a single intraperitoneal injection at Week 4 followed by PB mixed into each diet at a concentration of 0.05% from Week 6 to the final sacrifice at Week 12 when induction of GGT-positive foci was quantitatively analysed. On the ID and IS diets, respective numbers of GGT-positive foci were 6.3 and 14.2 cm-2. The sizes of foci were not altered by the iron content of the diet. The present results indicate that iron plays a role in the development of preneoplastic foci in the livers of rats initiated with DEN and promoted by PB especially in the initiation phase.


Br. J. Cancer (1991), 64, 839-842 © Macmillan Press Ltd., 1991

### Inhibitory effect of dietary iron deficiency on the induction of putative preneoplastic foci in rat liver initiated with diethylnitrosamine and promoted by phenobarbital

H. Yoshiji[1], D. Nakae[1], T. Kinugasa[1], M. Matsuzaki[2], A. Denda[1], T. Tsujii[3] & Y. Konishi[1]

[1]*Department of Oncological Pathology, Cancer Center, Nara Medical University, 840 Shijo-cho, Kashihara, Nara 634;* [2]*Research Division of SRL, Inc., 51 Komiya-cho, Hachi-ohji, Tokyo, 192; and* [3]*The Third Department of Internal Medicine, Nara Medical University, 840 Shijo-cho, Kashihara, Nara 634, Japan.*

**Summary** The effects of dietary iron deficiency on induction of putative preneoplastic, gamma-glutamyltransferase (GGT)-positive hepatocyte focal lesions in the liver of rats treated with diethylnitrosamine (DEN) followed by phenobarbital (PB) were investigated. Male Fischer 344 rats of 4 weeks old were placed on an iron deficient (ID) diet containing less than 5 p.p.m. of iron or an iron supplemented (IS) diet containing 180 p.p.m. of iron throughout experimental period of 12 weeks. Both groups of rats were administered 200 mg $kg^{-1}$ body weight of DEN by a single intraperitoneal injection at Week 4 followed by PB mixed into each diet at a concentration of 0.05% from Week 6 to the final sacrifice at Week 12 when induction of GGT-positive foci was quantitatively analysed. On the ID and IS diets, respective numbers of GGT-positive foci were 6.3 and 14.2 $cm^{-2}$. The sizes of foci were not altered by the iron content of the diet. The present results indicate that iron plays a role in the development of preneoplastic foci in the livers of rats initiated with DEN and promoted by PB especially in the initiation phase.

Recently, a role of iron in carcinogenic processes has attracted increasing attention. Ferric nitrilotriacetate has been revealed to exert renal carcinogenicity as well as nephrotoxicity (Li *et al.*, 1987; Hamazaki *et al.*, 1989; Umemura *et al.*, 1990). A role of iron in the growth of neoplastic cell population has also been pointed out (Hann *et al.*, 1988; Hann *et al.*, 1989; Hann *et al.*, 1990). It is thus conceivable that iron overload or deficiency in the target organ may alter the development of neoplasms. As far as iron overload is concerned, several results have been reported suggesting enhancing effects including a high incidence of hepatocellular carcinomas in human patients suffering from hereditary hemochromatosis has been reported (Bacon & Britton, 1989). In contrast, the effects of iron deficiency on carcinogenesis have still been the subject of only little attention.

Since it is generally accepted that hepatocyte foci demonstrating altered enzyme phenotypes including gamma-glutamyltransferase (GGT) expression are putative preneoplastic lesions for hepatocellular carcinomas, their quantitative analysis is a useful tool for evaluation of modulation of hepatocarcinogenesis in rats (Pitot & Sirica, 1980; Farber, 1984; Williams, 1989). In the present experiment, we examined the effects of dietary iron deficiency on induction of GGT-positive hepatocyte foci in rat liver initiated with diethylnitrosamine (DEN) and promoted by phenobarbital (PB) in order to cast further light on a role of iron in rat chemical carcinogenesis.

### Materials and methods

### *Animals*

Male Fischer 344 rats were obtained at 3 weeks of age from Japan SLC, Inc., Hamamatsu, Shizuoka, Japan and used after being acclimatised for 1 week with free access to Oriental MF Diet (Oriental Yeast Co. Ltd., Itabashi, Tokyo, Japan). The animals were housed in iron-free plastic cages in an air-conditioned room maintained at 25°C with a 12 h dark/light cycle and allowed access to food and tap water *ad libitum* throughout the investigation. The experiments were started by placing rats on the prepared diets after fasting fcr 18 h on the last day of their acclimation.

### *Chemicals and diets*

DEN was obtained from Wako Pure Chemical Industries, the City of Osaka, Osaka, Japan and diluted with 0.9% NaCl solution to 100 mg $ml^{-1}$. PB was supplied by Maruishi Pharmaceutical Co. Ltd., the City of Osaka, Osaka, Japan. Iron deficient (ID) and iron supplemented (IS) diets were synthesised by Oriental Yeast. The IS diet was designed to have a practically identical composition to Oriental MF Diet, a diet commonly used as a standard diet in numerous laboratories, including an iron content of 180 p.p.m. The ID diet was similarly produced with the exception that an iron content was diminished to less than 5 p.p.m. The ID or IS diet had a similar calorific content to Oriental MF Diet. Since using the IS diet in place of Oriental MF Diet did not alter any items analysed in the present experiments (data not shown), the IS diet is safely able to serve as a control diet.

### *Protocol for Experiment 1: Effects of dietary iron deficiency on induction of GGT-positive foci in rat liver*

The animals were divided into eight experimental groups based on the different treatments. Effective numbers are presented in Table I. Rats in Groups 1 to 4 received the ID diet throughout the experimental period of 12 weeks. Rats in Group 1 were given a single intraperitoneal injection of DEN at a dose of 200 mg $kg^{-1}$ body weight at Week 4, followed by administration of PB mixed into the ID diet at a concentration of 0.05% from Week 6 to Week 12. Rats in Group 2 received DEN without the following PB administration. Rats in Groups 3 and 4 were given the 0.9% NaCl solution vehicle instead of DEN with and without the following PB administration, respectively. Rats in Groups 5 to 8 received the IS diet throughout the experimental period. Rats in Groups 5 and 6 received DEN with and without the following PB administration, respectively. Rats in Groups 7 and 8 received the 0.9% NaCl solution in place of DEN with and without the following PB administration, respectively. All rats were sacrificed 12 weeks after the beginning of the experiment under ether anesthesia, and the livers were excised.

The livers were blotted, fixed in an ice-cold mixture of dehydrated ethanol and glacial acetic acid at a ratio of 19:1 for 3 h followed by an overnight incubation in 99.5% ethanol

Correspondence: D. Nakae, Department of Oncological Pathology, Cancer Center, Nara Medical University, 840 Shijo-cho, Kashihara, Nara 634, Japan.
Received 20 February 1991, and in revised form 10 June 1991.

**Table I** Experimental details and quantitative data regarding the influence of dietary iron deficiency on the induction of GGT-positive hepatocyte foci in the livers of rats initiated with DEN and promoted by PB

| Group | Diet | DEN | PB | Effective number of rats | Final body weight (g) | Liver weight (g $100^{-1}$) body weight | GGT-positive hepatocyte foci Number $cm^{-2}$ | GGT-positive hepatocyte foci Size (mean area; $mm^2 \times 10^2$) |
|---|---|---|---|---|---|---|---|---|
| 1 | ID | + | + | 9 | $272 \pm 11^{a,b}$ | $6.70 \pm 0.37^{c,d}$ | $6.3 \pm 3.6^{b,c}$ | $7.0 \pm 0.9$ |
| 2 | ID | + | – | 9 | $276 \pm 10$ | $5.77 \pm 0.23^{c}$ | $3.4 \pm 2.2^{b}$ | $7.4 \pm 0.8$ |
| 3 | ID | – | + | 9 | $294 \pm 14$ | $7.03 \pm 0.33^{c,d}$ | $0.7 \pm 0.5$ | $6.8 \pm 3.3$ |
| 4 | ID | – | – | 9 | $289 \pm 29$ | $6.02 \pm 0.36^{c}$ | $0.4 \pm 0.2$ | $6.8 \pm 1.5$ |
| 5 | IS | + | + | 8 | $269 \pm 13^{b}$ | $7.26 \pm 0.36^{d}$ | $14.2 \pm 4.4^{b,d}$ | $7.0 \pm 1.1$ |
| 6 | IS | + | – | 8 | $284 \pm 14$ | $6.55 \pm 0.56$ | $5.9 \pm 2.6^{b}$ | $7.4 \pm 0.7$ |
| 7 | IS | – | + | 8 | $299 \pm 11$ | $8.24 \pm 0.67^{d}$ | $0.5 \pm 0.2$ | $6.8 \pm 0.8$ |
| 8 | IS | – | – | 8 | $296 \pm 15$ | $6.99 \pm 0.75$ | $0.4 \pm 0.3$ | $6.7 \pm 1.5$ |

[a]Results are means ± standard deviations; [b]Significantly different from the respective groups not administered DEN (Groups 3, 4, 7 and 8 for Groups 1, 2, 5 and 6, respectively) ($P < 0.01$); [c]Significantly different from the respective iron-supplemented groups (Groups 5, 6, 7 and 8 for Groups 1, 2, 3 and 4, respectively) ($P < 0.01$); [d]Significantly different from the respective groups are administered PB (Groups 2, 4, 6 and 8 for Groups 1, 3, 5 and 7, respectively) ($P < 0.02$ in Liver Weight and $P < 0.001$ in Number $cm^{-2}$).

at 4°C and embedded in paraffin for histological and histochemical examinations of tissue sections. Two sequential liver sections were prepared from three major lobes of each liver sample, and one of them were processed routinely for hematoxylin and oesin stainings. In the other section, GGT activity was demonstrated histochemically by the method of Rutenberg *et al.* (1968). Quantitative analyses of the numbers and sizes of GGT-positive foci were performed as previously described (Denda *et al.*, 1989).

*Protocol for Experiment 2: Effects of dietary iron deficiency on blood biochemistry, liver contents of iron, cytochrome P450 and glutathione and peroxidative state of hepatocellular lipids in rats*

Groups 1 and 2, consisting of 35 rats each, respectively received the ID and IS diets for the first 4 weeks, followed by Oriental MF Diet for 2 weeks. During the experimental period of 6 weeks, sub-groups of five rats each from Groups 1 and 2 were serially sacrificed under ether anesthesia 0, 1, 2, 4, 4 3/7, 5 and 6 weeks after the commencement. On sacrifice, blood was taken from the bifurcation of the abdominal aorta, and the livers were excised. The blood was allowed to clot at room temperature and centrifuged for 20 min at 3,000 $\times g$ at 25°C to obtain serum. Aliquots of the blood obtained at Week 4 were immediately placed into test tubes with an anticoagulant.

The obtained sera were assayed for concentration of iron and total iron binding capacity (TIBC) by the method of Bonda (1968). Aliquots of the blood stored in the presence of an anticoagulant were determined for the numbers of red blood cells, the haemoglobin concentration and hematocrit score in a routine clinicobiochemical manner. The livers were taken, blotted, reduced to ashes by an overnight incubation at 100°C, dissolved in nitric acid and determined for iron content by atomic absorption spectrometry. From portions of the livers obtained at Week 4, microsomal fractions were prepared according to Curtis *et al.* (1984) and utilised for the determination of cytochrome P450 content by an adaptation from the ascorbate-phenazine ethosulfate method of Johannesen and DePierre (1978). The remaining portions of such livers were used for the assays of hepatic reduced glutathione (GSH) level and peroxidative state of hepatocellular lipids by adaptations from the methods of Sedlak and Lindsay (1968) and Yagi (1976), respectively. Protein concentration was determined by BCA Protein Assay employing bicinchoninic acid (Pierce Chemical Co., Rockford, IL, USA).

*Statistical analysis*

The statistical significance of intergroup differences in quantitative data was determined with the paired Student's *t*-test.

### Results

*Inhibitory effect of dietary iron deficiency on induction of GGT-positive foci in the liver of rats receiving DEN initiation followed by PB promotion (Experiment 1)*

Table I summarises final body and liver weights with the numbers and sizes of GGT-positive foci. During the experimental period, average food intake was constant among groups (data not shown). There were no significant differences among groups in the final body weight with the exception that, in the presence of PB-treatment, rats administered DEN were significantly lighter than those not administered DEN (compare Groups 1 and 3 and Groups 5 and 7). The liver of rats on the ID diet in respective treatment patterns was significantly lighter than that of rats on the IS diet (compare Groups 1 and 5, Groups 2 and 6, Groups 3 and 7 and Groups 4 and 8). On the other hand, the liver of rats administered PB in respective treatment patterns was significantly heavier than that of rats free from PB exposure (compare Groups 1 and 2, Groups 3 and 4, Groups 5 and 6 and Groups 7 and 8). In all of the groups, GGT-positive foci were developed. Those foci were histologically characterised as acidophilic foci with ground glass appearance constituted by more than 10 altered hepatocytes with hyperchromatic and enlarged nuclei containing prominent centrally located nucleoli according to the criteria described by Institute of Laboratory Animal Resources, National Research Council, National Academy of Sciences, Washington, DC (1980). In rats initiated with DEN, dietary iron deficiency significantly inhibited GGT-positive foci induction in the presence of PB promotion (compare Groups 1 and 5) and also exhibited a trend of inhibitory effect on the foci development in the absence of PB promotion (compare Groups 2 and 6). The sizes of GGT-positive foci were almost similar irrespective to treatment groups.

*Alteration of serum and hepatic status in rats by dietary iron deficiency (Experiment 2)*

Serum iron concentration of rats on the ID diet was depleted within 2 weeks, remaining low until 1 week after the rats were replaced on Ortiental MF Diet. Recovery of control level occurred in the following 1 week. Values in rats maintained on the IS diet did not change throughout the experimental period (Figure 1, left panel). The trend as a function of time for serum TIBC was completely opposite to that for serum iron concentration. Thus, TIBC increased in rats on the ID diet within 2 weeks, remained elevated until 1 week after the rats were replaced on Oriental MF Diet and recovered down to the control level in the next 1 week. The rats on the IS diet showed no change in TIBC values during

**Figure 1** Induction of reversible combined iron deficient anaemia and hepatic iron deficiency in male Fischer 344 rats of Experiment 2. Circles and open triangles represent the mean results for Groups 1 and 2, respectively (plus the positive standard deviations), of determinations for five individual rats. Closed squares represent respective values at the beginning of the experiment. Solid circles represent significant differences from the respective control values (at least $P < 0.05$). Arrows represent the timing of the replacement of the ID or IS diet to Oriental MF Diet.

the experimental period (Figure 1, middle panel). At Week 4, the numbers of red blood cells, haemoglobin concentration and haematocrit score were significantly lower in rats on the ID diet than in the IS diet animals (Table II). Hepatic iron content of rats on the ID diet became progressively depleted in a linear way after Week 1, the decrease being significant at Week 4. Depleted levels remained until 1 week after the rats were replaced on Oriental MF Diet and recovered up to the control level in the next 1 week. Placing rats on the IS diet did not alter their hepatic iron content (Figure 1, right panel). Cytochrome P450 content, GSH level or peroxidative state of hepatocellular lipids in the liver of rats was not altered significantly by feeding the ID diet for 4 weeks (Table III).

### Discussion

The present results clearly indicate an inhibitory effect of dietary iron deficiency on the development of GGT-positive preneoplastic focal lesions in the liver of rats initiated with DEN. Assuming a role of iron as an essential mineral required for growth and maintainance of life, however, the present results might be interpreted only to reflect a general growth retarding effect of dietary iron deficiency. Although liver weight of rats on the ID diet for 12 weeks was lighter than that of rats on the IS diet, dietary iron deficiency under the present experimental conditions did not exert a generalised growth retarding effect since growth of rats or liver enlargement resulting from the PB treatment was not affected. In the relatively rapid growing foci, however, a growth retarding effect of dietary iron deficiency might be amplified. Iron has been reported to play a role in the growth of neoplastic cell populations (Blatt & Stitely, 1987; Becton and Bryles, 1988; Hann *et al.*, 1988; Hann *et al.*, 1989; Hann *et al.*, 1990). Inhibitory effect of dietary iron deficiency on the induction of GGT-positive foci might, therefore, be ascribed to a reduction for the initiated cells to grow into preneoplastic foci by a reduced availability of iron.

The results in the present experiment, however, strongly suggest that iron plays a certain role in the initiation phase of chemical rat hepatocarcinogenesis. Thus, dietary iron deficiency reduced the number of foci developed in rat liver without an alteration on their size. According to the well accepted description of Pitot *et al.* (1989), number and size of foci indicate initiating and promoting activities, respectively. It is, therefore, unlikely that inhibitory effect of dietary iron deficiency on development of GGT-positive foci is fully attributable to its growth retarding effect. In addition, a preceeding 4-week dietary iron deficiency did not seem to affect a bioactivation of DEN because of a lack of an alteration of liver content of cytochrome P450, relating to the bioactivation of DEN (Ton & Fong, 1984; Ioannides & Parke, 1987), although an overall content of cytochrome P450 may not be sufficient to address the issue of potential effects on DEN bioactivation. Accumulating evidences have suggested a participation of an oxidative stress in chemical carcinogenesis in its initiation, promotion and progression phases (Slaga *et al.*, 1981; Copeland, 1983; Cerutti, 1985; O'Connel *et al.*, 1986; Marnett, 1987; Vuillaume, 1987), and iron is well known to be one of the most essential catalysts in oxidative stress reactions to generate highly reacting activated oxygen species such as hydroxyl radical (Halliwell & Gutteridge, 1984). A causal participation of an iron-associated oxidative stress is postulated in the mechanisms of the high risk of hepatocellular carcinoma development in human patients suffering from hereditary hemochromatosis (Bacon & Britton, 1989) and of the renal carcinogenicity, especially its initiation phase, as well as nephrotoxicity of ferric nitrilotriacetate in mice and rats (Hamazaki *et al.*, 1989; Umemura *et*

**Table II** Anaemia induced in rats on the ID diet for 4 weeks

| *Group* | *Diet* | *Number of red blood cells* ($10^4\ \mu l^{-1}$) | *Haemoglobin concentration* ($mg\ ml^{-1}$) | *Haematocrit score* (%) |
|---|---|---|---|---|
| 1 | ID | $731 \pm 11^{a,b}$ | $130 \pm 10^{b}$ | $34 \pm 3^{b}$ |
| 2 | IS | $855 \pm 17$ | $150 \pm 0$ | $44 \pm 1$ |

[a]Results are means ± standard deviations of determinations for five individual rats; [b]Significantly lower than Group 2 value ($P < 0.05$).

Table III Cytochrome P450 content, GSH level and peroxidative state of hepatocellular lipids in the livers of rats on the ID diet for 4 weeks

| *Group* | *Diet* | *Cytochrome P450 content (pmol mg$^{-1}$ protein)* | *GSH level (μg mg$^{-1}$ protein)* | *Peroxidative state of hepatocellular lipids (nmol MDA equivalent g$^{-1}$ wet liver)* |
|---|---|---|---|---|
| 1 | ID | 366 ± 69[a] | 9.5 ± 0.8 | 1.2 ± 0.1 |
| 2 | IS | 323 ± 39 | 9.4 ± 0.8 | 1.0 ± 0.3 |

[a]Results are means ± standard deviations of determinations for five individual rats. MDA; malondialdehyde.

al., 1990). In the recent review, Bartsch et al. (1989) described that dimethylnitrosamine and other nitrosamines may be activated into DNA binding intermediates by cytochrome P450-dependent formation of alfa-nitrosamino radicals or photochemically, drew pathways for activation of dialkylnitrosamines involving free radical intermediates and suggested that free radicals damage and DNA alkylation are involved in carcinogenesis induced by nitrosamines. Although it has not yet elucidated whether free radicals also participate in the metabolism of DEN in particular, Sholtz et al. (1990) observed the increased levels of reactive oxygen formation in neoplastic liver nodules in rats initiated with DEN. In this context, the inhibitory effect of dietary iron deficiency on development of preneoplastic foci in rat liver initiated with DEN may be due to the reduced initiation resulting from a deficiency of catalytic iron required in oxidative stress reactions.

In conclusion, the present results indicate that dietary iron deficiency exerted an inhibitory effect on the induction of putative preneoplastic hepatocyte focal lesions in rat liver initiated with DEN chiefly by a reduction of DEN initiation. Inhibitory effects on the growth and development of the foci, however, may also influence the inhibition of foci induction by dietary iron deficiency.

This work was supported by Grants-in-Aid for Cancer Research (1-22) and for the Comprehensive Ten-Year Strategy for Cancer Control, both from the Ministry of Health and Welfare of Japan.

### References

BACON, B.R. & BRITTON, R.S. (1989). Hepatic injury in chronic iron overload, role of lipid peroxidation. *Chem. Biol. Interact.*, **70**, 183.

BARTSCH, H., HIETANEN, E. & MALAVEILLE, C. (1989). Carcinogenic nitrosamines: free radical aspects of their action. *Free Radical Biol. Med.*, **7**, 637.

BECTON, D.L. & BRYLES, P. (1988). Deferoxamine inhibition of human neuroblastoma viability and proliferation. *Cancer Res.*, **48**, 7189.

BLATT, J. & STITELY, S. (1987). Anti-neuroblastoma activity of desferoxamine in human cell lines. *Cancer Res.*, **47**, 1749.

BONDA, J. (1968). Determination of iron with bathophenanthrolone without deproteination. *Clin. Chim. Acta*, **21**, 159.

CERUTTI, P. (1985). Prooxidant states and tumor promotion. *Science*, **227**, 375.

COPELAND, E.S. (1983). Free radicals in promotion. A chemical pathology study section report. *Cancer Res.*, **43**, 5631.

CURTIS, M.T., GILFOR, D. & FARBER, J.L. (1984). Cytochalasin delays but does not prevent the cell death from anoxia. *Arch. Biochem. Biophys.*, **235**, 644.

DENDA, A., URA, H., TSUJIUCHI, T. & 6 others (1989). Possible involvement of arachidonic acid metabolism in phenobarbital promotion of hepatocarcinogenesis. *Carcinogenesis*, **10**, 1929.

FARBER, E. (1984). The multistep nature of cancer development. *Cancer Res.*, **44**, 4217.

HALLIWELL, B. & GUTTERIDGE, J.M.C. (1984). Oxygen toxicity, oxygen radicals, transition metals and disease. *Biochem. J.*, **219**, 1.

HAMAZAKI, S., OKADA, S., LI, J.-L., TOYOKUNI, S. & MIDORIKAWA, O. (1989). Oxygen reduction and lipid peroxidation by iron chelates with special reference to ferric nitrilotriacetate. *Arch. Biochem. Biophys.*, **272**, 10.

HANN, H.L., STAHLHUT, M.W. & BLUMBERG, B.S. (1988). Iron nutrition and tumor growth. Decreased tumor growth in iron deficient mice. *Cancer Res.*, **48**, 4168.

HANN, H.L., STAHLHUT, M.W. & HANN, C.L. (1990). Effect of iron and desferoxamine on cell growth and *in vitro* ferritin synthesis in human hepatoma cell lines. *Hepatology*, **11**, 566.

HANN, H.L., STAHLHUT, M.W., MENDUKE, H., LONDON, W.T. & BLUMBERG, B.S. (1989). Iron nutrition and tumor growth. Observation in spontaneous mammary tumors in mice. *Proc. Am. Soc. Clin. Oncol.*, **8**, 59.

INSTITUTE OF LABORATORY ANIMAL RESOURCES, NATIONAL RESEARCH COUNCIL, NATIONAL ACADEMY OF SCIENCES, WASHINGTON, D.C. (1980). Histologic typing of liver tumors of the rat. *J. Natl Cancer Inst.*, **64**, 177.

IOANNIDES, C. & PARKE, D.V. (1987). The cytochrome P-448. A unique family of enzymes involved in chemical toxicity and carcinogenesis. *Biochem. Pharmacol.*, **36**, 4197.

JOHANNESEN, K.A.M. & DEPIERRE, J.W. (1978). Measurement of cytochrome *P*-450 in the presence of large amounts of contaminating hemoglobin and methemoglobin. *Anal. Biochem.*, **86**, 725.

LI, J.-L., OKADA, S., HAMAZAKI, S., EBINA, Y. & MIDORIKAWA, O. (1987). Subacute nephrotoxicity and induction of renal cell carcinoma in mice treated with ferric nitrilotriacetate. *Cancer Res.*, **47**, 1867.

MARNETT, L.J. (1987). Peroxyl free radicals. Potential mediators of tumor initiation and promotion. *Carcinogenesis*, **8**, 1365.

O'CONNEL, J.F., KLEIN-SZANTO, A.J.P., DIGIOVANNI, D.M., FRIES, J.W. & SLAGA, T.J. (1986). Enhanced malignant progression of mouse skin tumors by the free-radical generator benzoyl peroxide. *Cancer Res.*, **46**, 2863.

PITOT, H.C., CAMPBELL, H.A., MARONPOT, R. & 6 others (1989). Critical parameters in the quantitation of the stages of initiation, promotion and progression in one model of hepatocarcinogenesis in the rat. *Toxicol. Pathol.*, **17**, 594.

PITOT, H.C. & SIRICA, A.E. (1980). The stages of initiation and promotion in hepatocarcinogenesis. *Biochim. Biophys. Acta*, **605**, 191.

RUTENBERG, A.M., KIM, H., FISCHBEIN, J., HAUKER, J.S., WASSERKRUG, H.C. & SELIGMAN, R. (1968). Histochemical and ultrastructural demonstration of gamma-glutamyltranspeptidase activity. *J. Histochem. Cytochem.*, **17**, 517.

SCHOLZ, W., SCHUTZE, K., KUNZ, W. & SCHWARZ, M. (1990). Phenobarbital enhances the formation of reactive oxygen in neoplastic rat liver nodules. *Cancer Res.*, **50**, 7015.

SEDLAK, J. & LINDSAY, R.H. (1968). Elimination of total, protein bound, and nonprotein sulfhydryl groups in tissue with Ellman's Reagent. *Anal. Biochem.*, **25**, 192.

SLAGA, T.J., KLEIN-SZANTO, A.J.P., TRIPLETT, L.L., YOTTI, L.P. & TROSKO, J.E. (1981). Skin tumor promoting activity of benzoyl peroxide, a widely used free radical generating compound. *Science*, **213**, 1023.

TON, C.C.T. & FONG, L.Y.Y. (1984). The effects of ascorbic acid deficiency and excess on the metabolism and toxicity of N-nitrosodimethylamine and N-nitrosodiethylamine in the Guinea pig. *Carcinogenesis*, **5**, 533.

UMEMURA, T., SAI, K., TAKAGI, A., HASEGAWA, R. & KUROKAWA, Y. (1990). Formation of 8-hydroxydeoxyguanosine (8-OH-dG) in rat kidney DNA after intraperitoneal administration of ferric nitrilotriacetate (Fe-NTA). *Carcinogenesis*, **11**, 345.

VUILLAUME, M. (1987). Reduced oxygen species, mutation induction and cancer initiation. *Mutat. Res.*, **186**, 43.

WILLIAMS, G.M. (1989). The significance of chemically-induced hepatocellular altered foci in rat liver and application to carcinogen detection. *Toxicol. Pathol.*, **17**, 663.

YAGI, K. (1976). A simple fluorometric assay for lipoperoxide in blood plasma. *Biochem. Med.*, **15**, 212.